# Preoperative Endoscopic Ultrasound-Guided Hepaticogastrostomy Facilitates Decompression and Diagnosis in Patients With Suspected Malignant Biliary Obstruction: A Case Series

**DOI:** 10.7759/cureus.23209

**Published:** 2022-03-16

**Authors:** Nicholas J Koutlas, Ella M LePage, Tomas Lucioni, Swati Pawa, Rishi Pawa

**Affiliations:** 1 Gastroenterology, Atrium Health Wake Forest Baptist, Winston-Salem, USA; 2 Internal Medicine, Atrium Health Wake Forest Baptist, Winston-Salem, USA

**Keywords:** whipple procedure, malignant biliary obstruction, lumen apposing metal stent, hepaticogastrostomy, endoscopic ultrasound

## Abstract

Endoscopic ultrasound-guided hepaticogastrostomy (EUS-HG) is increasingly being used as an alternative to percutaneous transhepatic biliary drainage (PTBD) after unsuccessful endoscopic retrograde pancreatography (ERCP). This technique has also been utilized for diagnosis of malignant biliary obstruction by providing biliary access for antegrade cholangioscopy with biopsies and brushings for cytology and fluorescent in situ hybridization (FISH). However, the potential impact of EUS-HG on surgical candidacy in cases with resectable disease remains unknown. We present three patients who underwent pancreaticoduodenectomy (Whipple procedure) for suspected distal malignant biliary obstruction following EUS-HG. Biliary drainage was achieved in all three patients preoperatively and a diagnosis of malignancy could be established in two of the three cases using this technique. There were no procedure-related complications. The HG metal stent was removed eight weeks post-operatively with cholangiogram showing a patent hepaticojejunostomy in all three patients. Mean length of follow-up after EUS-HG was 298 +/- 96 days. Our case series demonstrates that EUS-HG is an effective method to achieve biliary decompression in patients with an inaccessible papilla. The mature HG tract can subsequently be used to obtain a tissue diagnosis. Lastly, EUS-HG does not preclude patients from undergoing a curative Whipple procedure.

## Introduction

In patients with malignant biliary obstruction, endoscopic retrograde pancreatography (ERCP) is the current standard of care for biliary decompression. However, ERCP is unsuccessful in 5-10% of cases due to surgically altered anatomy or tumor invasion causing ampullary distortion or gastric outlet obstruction [1]. In this subset of patients, percutaneous transhepatic biliary drainage (PTBD) has traditionally been the most widely utilized option. However, PTBD has been associated with significant morbidity from complications such as bleeding, infections including insertion site cellulitis and cholangitis, tube dislodgement, pancreatitis, and pneumothorax. Additionally, the presence of an external drain causes patient discomfort and negatively impacts quality of life [2,3]. 

Endoscopic ultrasound-guided hepaticogastrostomy (EUS-HG) is emerging as a suitable alternative to PTBD in patients after failed attempt at ERCP. EUS-HG has been associated with similarly high technical and clinical success rates with fewer reinterventions when compared to PTBD [2]. In addition to biliary decompression, EUS-HG can provide diagnostic information by facilitating tissue acquisition. Once EUS-HG has been completed, it can be used as a port for performing antegrade cholangioscopy with intraductal biopsies and brushings for cytology and fluorescent in situ hybridization (FISH). Despite its apparent advantages, concerns exist about the potential impact of EUS-HG on surgical candidacy. Upon review of the current literature, there has been one case described of a patient with history of Roux-en-Y gastric bypass surgery who presented with obstructive jaundice from a pancreatic head mass. EUS-HG was successful in achieving biliary decompression and a tissue diagnosis prior to a successful Whipple procedure [4]. 

In this case series, we describe EUS-HG in three patients with suspected malignant biliary obstruction after unsuccessful ERCP. EUS-HG achieved biliary decompression and assisted with tissue diagnosis but did not preclude future surgical intervention.

## Case presentation

Procedure detail

**Figure 1:**
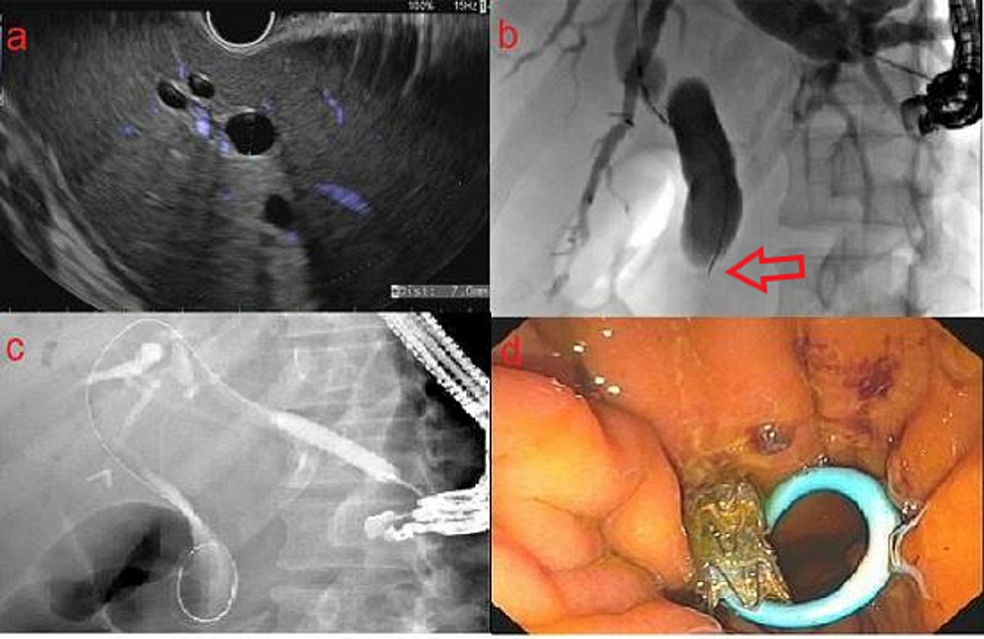
Creation of the hepaticogastrostomy tract using endoscopic ultrasound guidance. a: EUS demonstrating dilated segment 3 biliary radicle. b: Antegrade cholangiogram demonstrating dilated intra and extrahepatic ducts with high grade stricture (red arrow) in the distal common bile duct. c: Dilation of hepaticogastrostomy tract using a Hurricane biliary dilating balloon. d: Successful placement of metal hepaticogastrostomy stent with plastic double pigtail biliary stent anchoring it in place.

All EUS procedures were performed by a single endosonographer using a linear echoendoscope (GF-UCT180; Olympus, Center Valley, PA, USA). A dilated segment 3 biliary radicle was identified from the lesser curvature of the stomach and punctured with a 19-gauge fine needle aspiration (FNA) needle (EchoTip Ultra HD; Cook Medical, Winston-Salem, NC, USA). Contrast was injected resulting in opacification of the biliary tree. A 0.025 inch in diameter and 450 cm in length straight tip VisiGlide 2 wire (Olympus) was advanced past the left hepatic duct into the distal bile duct and the small bowel. The HG tract was dilated using a 6 mm x 4 cm Hurricane biliary dilating balloon (Boston Scientific, Marlborough, MA, USA) followed by placement of an 8 mm x 80 mm fully covered, self-expandable, and non-foreshortening Gore Viabil biliary stent (W.L. Gore Associates, Flagstaff, AZ, USA). The metal stent was anchored using a 7 Fr x 18 cm double pigtail plastic biliary stent with the proximal end in the stomach and the distal end past the ampulla and in the small bowel (Figure 1). All patients were discharged home following EUS-HG with a follow-up appointment scheduled in two weeks for ERCP through the HG tract. 

Case 1

A 37-year-old male with prior history of cholelithiasis and choledocholithiasis treated with biliary sphincterotomy, balloon sweeps, and subsequent cholecystectomy presented with painless jaundice. Initial labs are noted in Table 1. 

**Table 1:** Pertinent lab values prior to and after EUS-HG. EUS-HG: Endoscopic ultrasound guided hepaticogastrostomy, ALP: Alkaline phosphatase, AST: Aspartate aminotransferase, ALT: Alanine aminotransferase

	Total Bilirubin pre EUS-HG (mg/dL)	Total Bilirubin two weeks post EUS-HG (mg/dL)	Initial ALP (IU/L)	ALP two weeks post EUS-HG(IU/L)	Initial ALT (IU/L)	ALT two weeks post EUS-HG (IU/L)	Initial AST (IU/L)	AST two weeks post EUS-HG (IU/L)	CA 19-9 (U/mL)
Case 1	10.3	1.0	2180	151	152	57	182	35	3.1
Case 2	7.9	2.4	868	264	292	41	200	41	169.4
Case 3	1.8	0.4	168	115	176	17	249	23	413.8
Reference range	0.1-1.2	0.1-1.2	25-125	25-125	5-50	5-50	5-40	5-40	0-35

**Figure 2:**
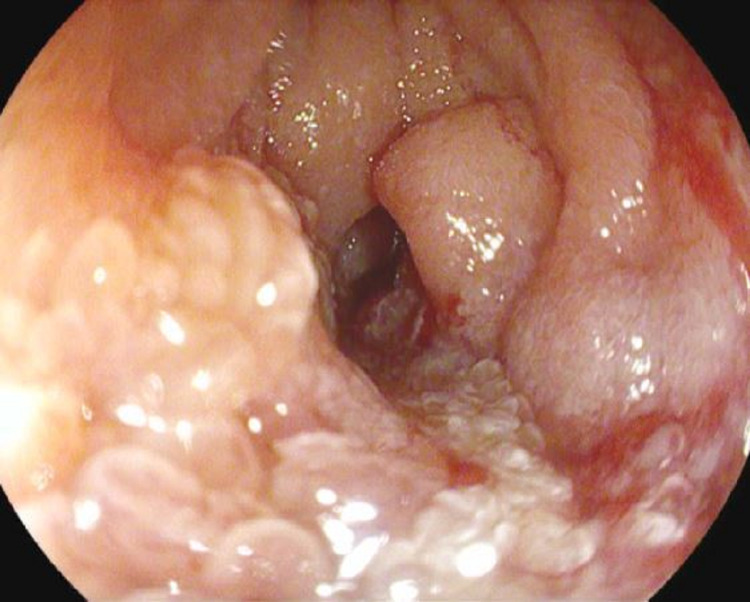
Edematous mucosa in the duodenal sweep resulting in luminal narrowing.

Contrast enhanced computed tomography (CT) scan demonstrated slightly increased intra- and extrahepatic biliary ductal dilation compared to imaging one year prior. On ERCP, narrowing of the duodenal sweep was visualized with abnormal appearing duodenal mucosa (Figure 2). The duodenoscope was advanced into the second portion of the duodenum after balloon dilation of a duodenal stricture. However, the major papilla could not be located. Duodenal biopsies were obtained with cold forceps and were negative for malignancy. 

**Figure 3:**
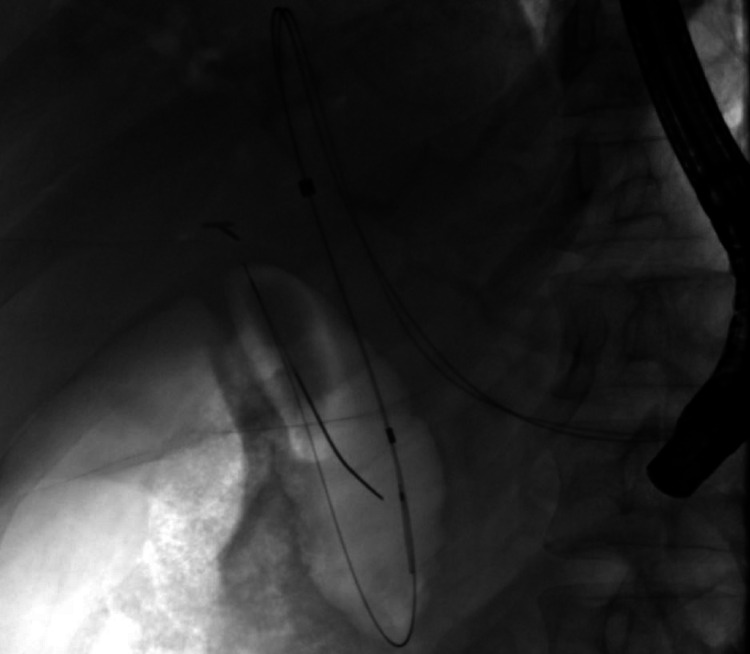
Antegrade brushings being obtained for cytology and fluorescent in situ hybridization.

EUS-HG was then performed according to the above description. Cholangiogram on follow-up ERCP two weeks after EUS-HG showed a 10 mm long stricture in the distal common bile duct (CBD). Cholangioscopy (SpyScope DS; Boston Scientific) was performed antegrade through the HG tract and a high-grade stricture was visualized in the distal CBD with evidence of neovascularization. SpyBite forceps (Boston Scientific) were unable to be advanced to the level of the stricture due to the acute angulation of the cholangioscope in the intrahepatic duct. Brushings were performed for cytology and FISH (Figure 3).

**Figure 4:**
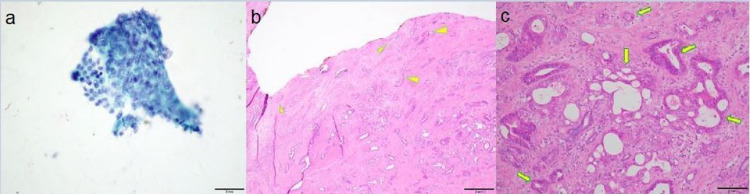
a: Cytology notes rare groups of highly atypical glandular cells. b: Surgical pathology specimen confirms invasive adenocarcinoma (arrowheads) involving the common bile duct. c: Moderately to poorly differentiated adenocarcinoma consistent with pancreatobiliary type (arrows).

Cytology showed rare groups of highly atypical glandular cells, suspicious for adenocarcinoma. FISH was positive for malignancy of the pancreatobiliary tract. Patient then underwent successful pancreaticoduodenectomy (Whipple procedure) with simultaneous removal of plastic HG stent 55 days after EUS-HG. Surgical pathology confirmed extrahepatic cholangiocarcinoma (stage IIIA; T2 N2 M0) (Figure 4).

**Figure 5:**
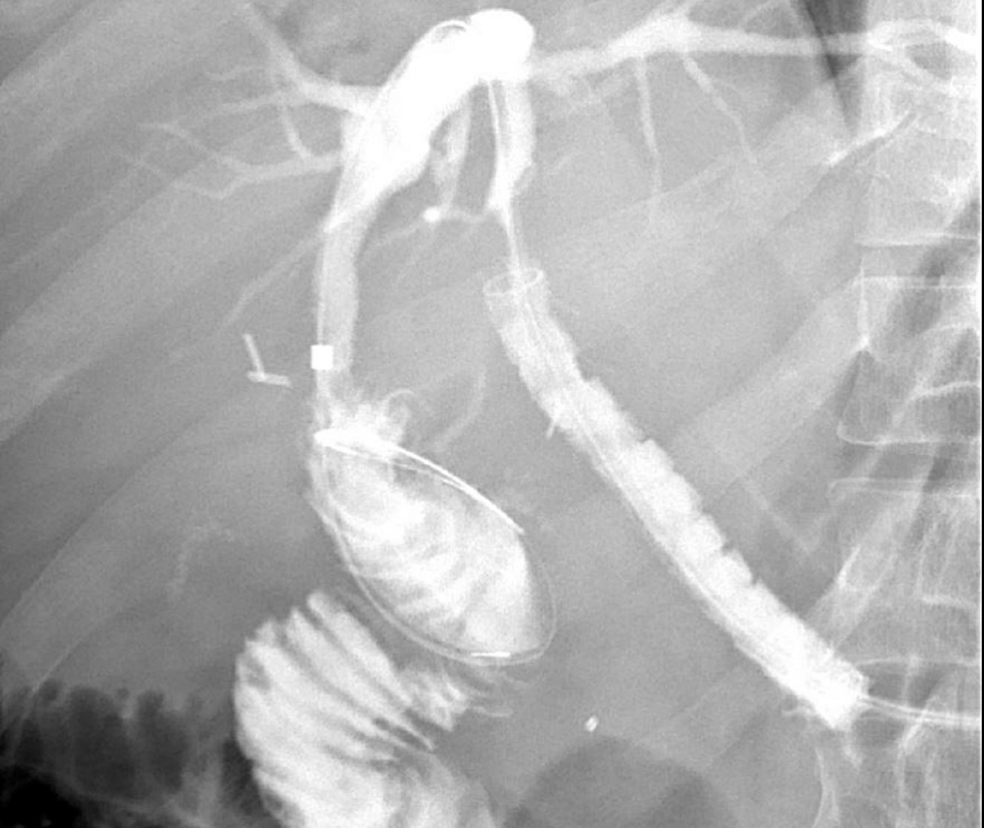
Cholangiogram via the hepaticogastrostomy tract demonstrating patent hepaticojejunostomy after Whipple procedure.

Follow-up ERCP was performed at eight weeks after the Whipple procedure. Cholangiogram through the HG tract showed a patent hepaticojejunostomy (Figure 5). 

The HG metal stent was removed and was not replaced. He subsequently was treated with adjuvant chemotherapy and radiation. Patient has had follow-up for 405 days after EUS-HG and is doing well clinically. 

Case 2

**Figure 6:**
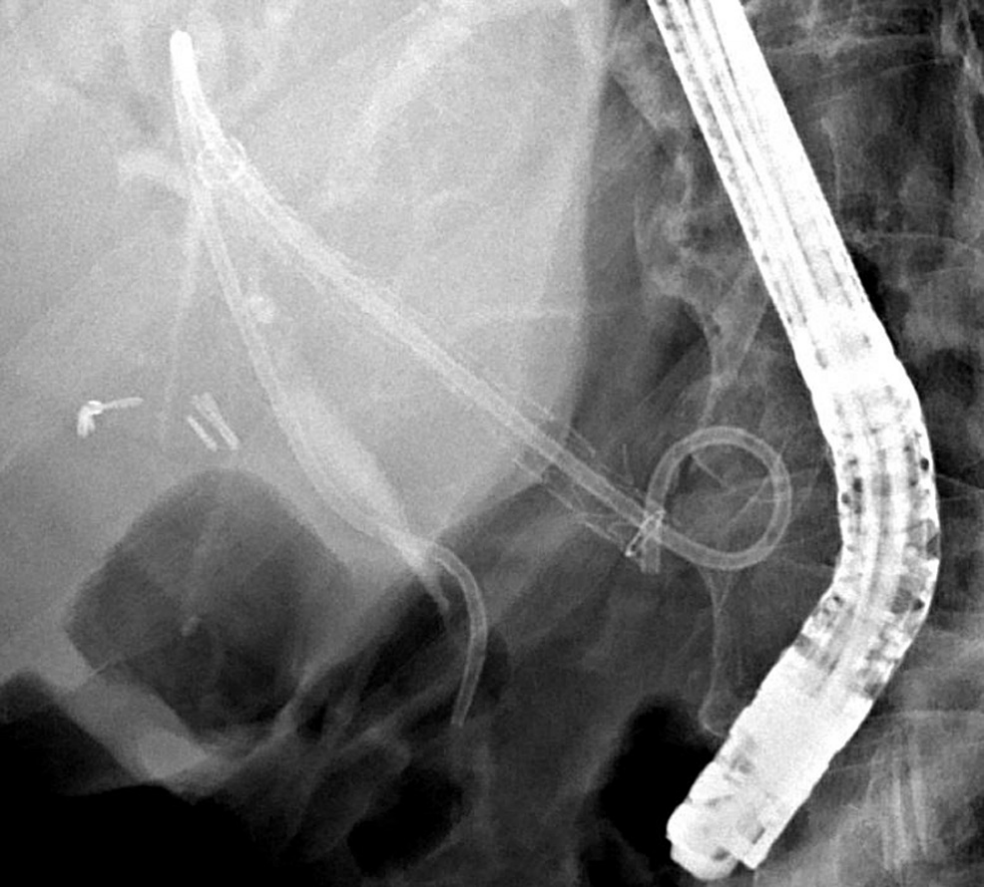
Fluoroscopic image showing placement of hepaticogastrostomy stents for biliary drainage.

A 48-year-old female with history of obesity, chronic obstructive pulmonary disease (COPD), and prior cholecystectomy presented to hepatology clinic for evaluation of abnormal liver enzymes. Pertinent labs are noted in Table 1. Abdominal ultrasound was notable for a mildly dilated CBD up to 12 mm. A magnetic resonance cholangiopancreatography (MRCP) was obtained and showed mild dilation of the CBD thought to be related to prior cholecystectomy. Serologic workup was negative. Liver biopsy demonstrated hepatocellular and canalicular cholestasis with mild nonspecific portal and lobular inflammation with bile duct injury. A diagnosis of drug-induced liver injury secondary to Bactrim was made. Follow-up labs six months later showed a persistently elevated total bilirubin. Given failure of normalization of liver enzymes despite withholding the offending drug, a CT scan was obtained. This showed intrahepatic and extrahepatic biliary dilatation with a 3 mm stone in the distal CBD. Mildly distended proximal duodenum with mild wall thickening of the second and third portion of the duodenum was also noted with surrounding inflammatory changes. On ERCP, edematous and congested duodenal mucosal folds were seen prohibiting visualization of the major papilla. Duodenal biopsies did not show any evidence of tumor infiltration. On EUS, a 13.1 mm x 14.9 mm ill-defined, hypoechoic, and heterogeneous in echotexture area was seen in the head of the pancreas. A 22G FNA needle (Cook Medical) was used to perform aspiration of this lesion but the specimen was non-diagnostic. An EUS-HG was subsequently performed for biliary drainage (Figure 6).

**Figure 7:**
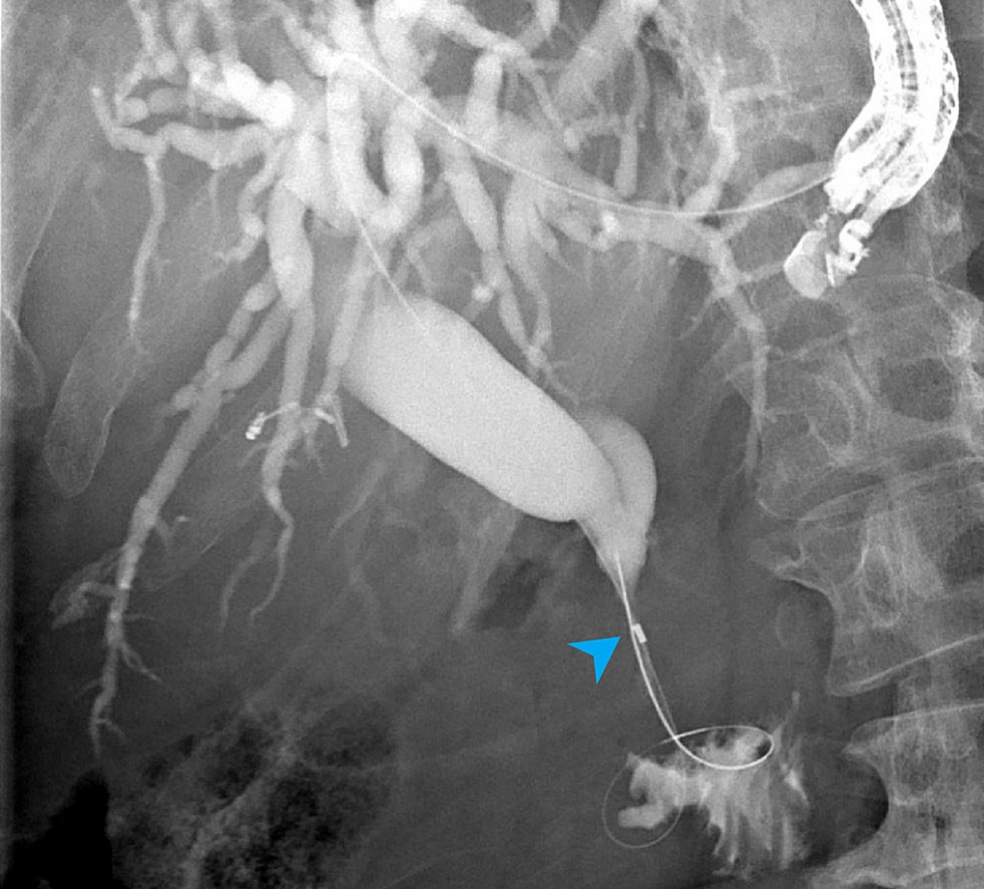
Cholangiogram showing a dilated biliary tree with distal bile duct stricture (blue arrowhead).

Two weeks post EUS-HG, patient returned for cholangiography and cholangioscopy through the HG tract. Cholangiography showed a high-grade stricture in the distal CBD with upstream ductal dilation (Figure 7).

**Figure 8:**
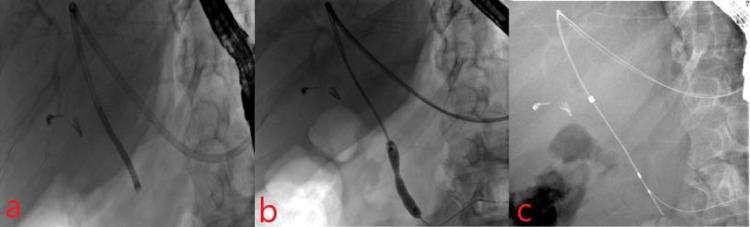
a: Cholangioscope advanced via the hepaticogastrostomy tract to obtain biopsies from distal bile duct stricture. b: Balloon dilation of distal bile duct stricture. c: Antegrade brushings for cytology and fluorescent in situ hybridization.

Cholangioscopy with intraductal biopsies and brushings for cytology and FISH were performed and were suggestive of malignancy (Figure 8). 

Patient eventually underwent successful Whipple procedure with surgical pathology examination confirming locally advanced pancreatic adenocarcinoma (T1cN2). The HG metal stent was removed eight weeks post-Whipple without need for replacement. She was started on adjuvant chemotherapy post-surgery and is clinically doing well after 269 days of follow-up. 

Case 3

**Figure 9:**
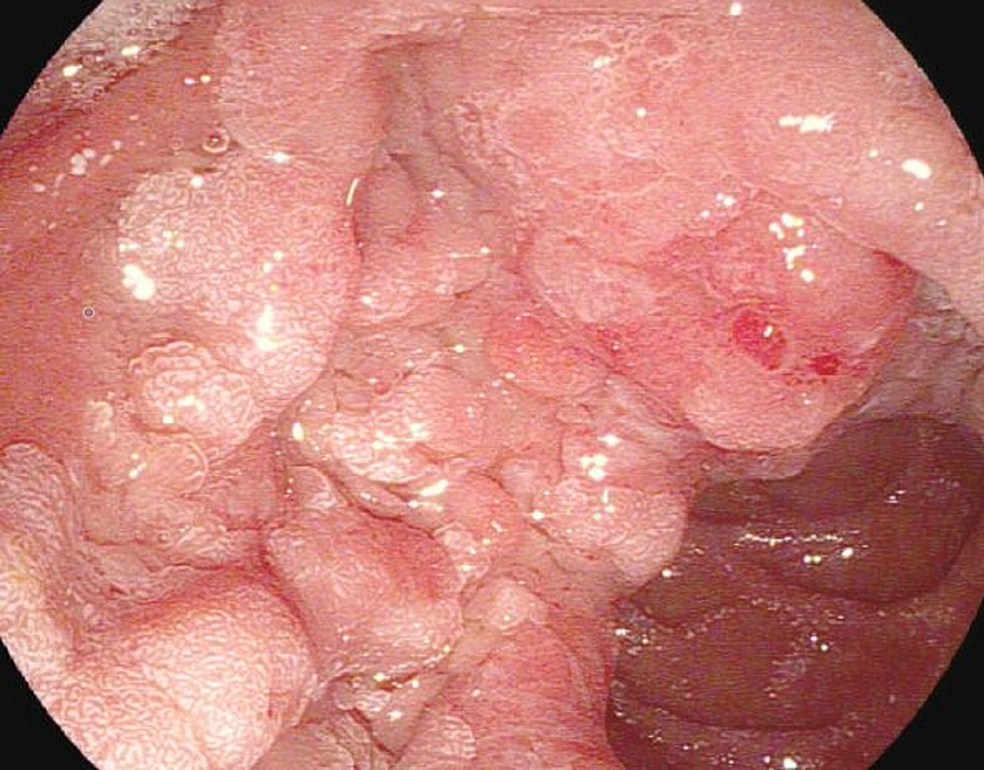
Peri-ampullary mass seen in the second portion of the duodenum precluding biliary cannulation.

An 81-year-old female with history of COPD presented for evaluation of incidentally found abnormal liver enzymes. Labs are shown in Table 1. Contrast enhanced CT scan noted marked extra and intrahepatic bile duct dilation with a mass-like density within the peri-ampullary portion of the CBD. On ERCP, a large peri-ampullary mass measuring 4 cm in size was seen and precluded bile duct cannulation (Figure 9).

**Figure 10:**
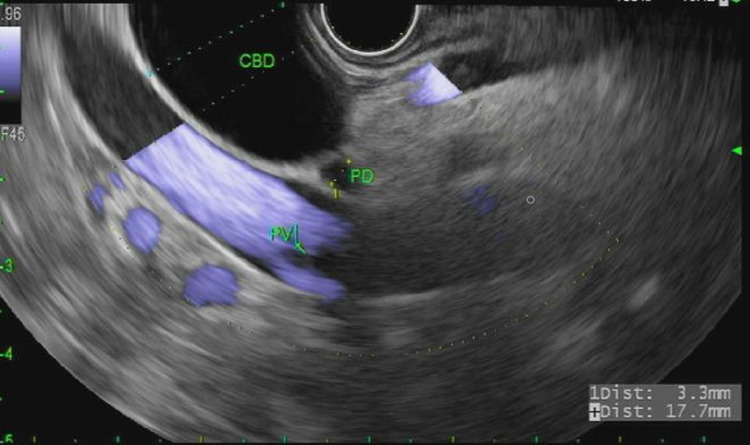
Endoscopic ultrasound showing dilated common bile duct in the head of the pancreas. CBD: common bile duct, PD: pancreatic duct, PV: portal vein

Cold forceps biopsies of the mass later revealed an adenoma with no evidence of malignancy. On EUS, the common bile duct was dilated with extension of mass lesion into the distal CBD and pancreatic duct (PD) for more than 1 cm (Figure 10).

**Figure 11:**
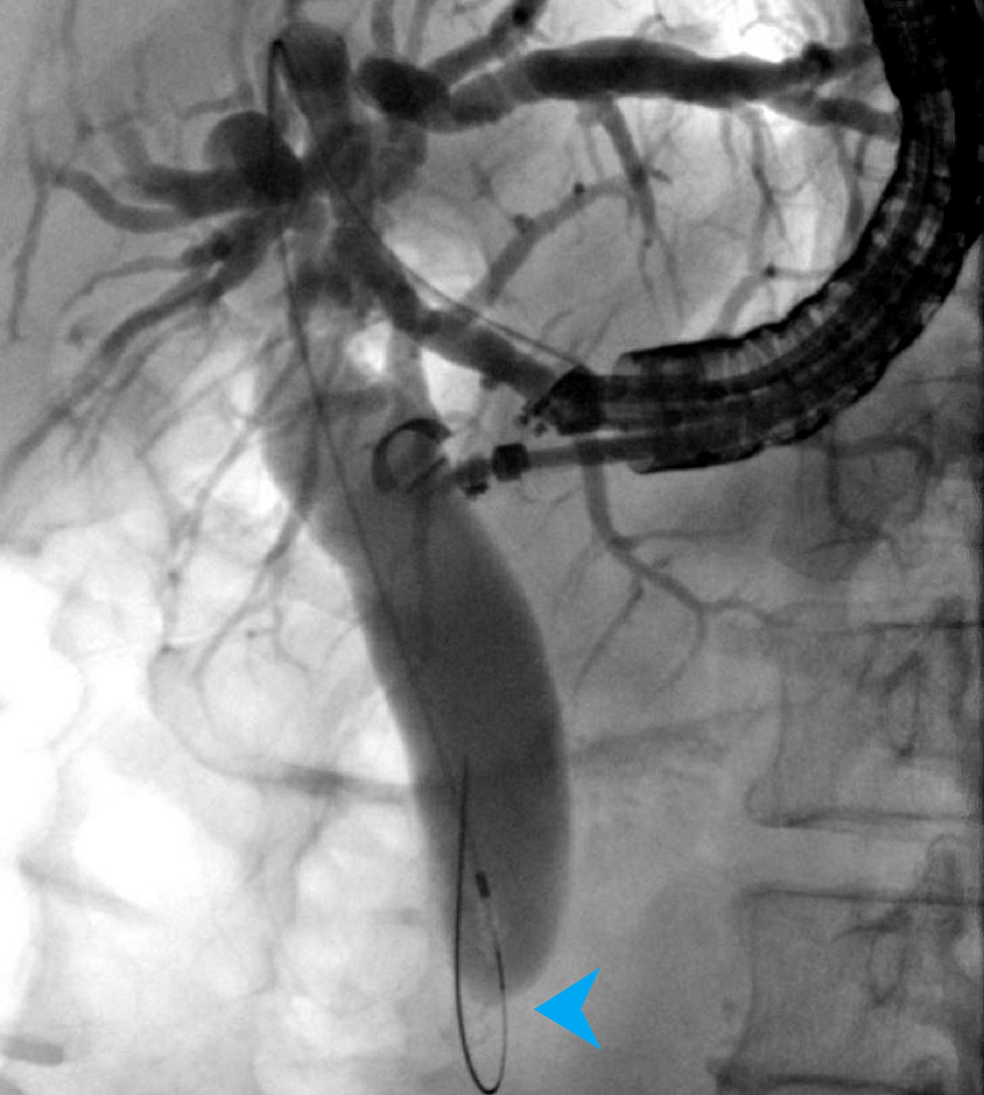
Cholangiogram showing a dilated biliary tree with distal bile duct stricture (blue arrowhead).

The patient underwent successful EUS-HG in the same session without complication. Cholangiogram showed a dilated CBD measuring 17 mm with a high-grade stricture in the distal CBD (Figure 11). 

Given the size of the ampullary adenoma with extension into the distal CBD and PD, a decision was made to proceed with surgical management without the need for further diagnostic evaluation. She had a successful pylorus-preserving Whipple procedure 37 days after EUS-HG. Surgical pathology confirmed an ampullary tubulovillous adenoma with complete excision. After surgery, the HG metal stent was removed without necessitating replacement. Patient remains in good clinical condition after 219 days of follow-up. 

## Discussion

ERCP can be technically unfeasible in patients with surgically altered gastrointestinal anatomy or tumor invasion causing distortion of the ampulla or gastric outlet obstruction. Traditionally, these patients have been managed primarily with percutaneous drainage. While PTBD has proven efficacious at achieving biliary decompression, it is burdened by substantial risk for adverse events and negative impact on patient quality of life [2,3,5]. 

EUS-guided biliary drainage (EUS-BD) was first described in 2001 by Giovannini et al. as an endoscopic option for these patients [6]. This term includes three separate techniques for biliary access: EUS-guided rendezvous, EUS-guided choledochoduodenostomy (EUS-CD), and EUS-guided hepaticogastrostomy. A study by Khashab et al. comparing rendezvous drainage with transluminal drainage (EUS-HG and EUS-CD) showed similar rates of technical success, clinical success, and adverse events [7]. Despite this, each technique has its own advantages and disadvantages. EUS-guided rendezvous is accomplished without the need for the creation of a biloenteric fistula, reducing the risk of pneumoperitoneum. However, it has been associated with increased fluoroscopy times due to difficulty in passing the guidewire through the papilla [2,5,7]. It also requires access to the papilla to be successful. EUS-CD has been shown to be as safe and effective as EUS-HG [8]. However, it is often not an option in cases with surgically altered anatomy or gastric outlet obstruction [2,5]. 

EUS-HG was first utilized for management of malignant biliary obstruction in 2003 [9]. With the development of novel devices and refinement of techniques in therapeutic EUS, EUS-HG has become an effective and safe option for decompression in these patients. In a retrospective analysis by Imai et al., technical success and clinical success were achieved in 97.6% and 90.2% of the 37 patients included in the study [10]. Additionally, another retrospective study of 41 patients undergoing EUS-HG reported a 90.2% technical success rate [11]. In regard to adverse events, the rate of complications with EUS-HG reportedly is about 15-20% with notable complications including infection, bleeding, pneumoperitoneum, bile leak, and stent migration [12]. Importantly, when compared with PTBD, EUS-HG has been found to have equally high technical and clinical success rates with a similar frequency of adverse events [2]. 

While the initial indication for EUS-HG was palliation of malignant biliary obstruction, its applications have since broadened to include treatment of benign biliary diseases. The HG tract can serve as a conduit for performing antegrade treatment of biliary lithiasis, benign strictures, and bile leaks [3]. Stone removal with balloon catheter, mechanical lithotripsy, and cholangioscopy with electrohydraulic lithotripsy (EHL) has been reported [13,14]. In a study of nine patients with Roux-en-Y gastric bypass anatomy and biliary lithiasis, stone removal was achieved in all nine cases, four of which required cholangioscopy with EHL [15]. In a larger retrospective study of 29 patients with surgically altered anatomy and choledocholithiasis, 21 had successful stone removal via the HG tract [16]. Antegrade deployment of metal biliary stents into the distal common bile duct for benign strictures has also been described [3].

In addition to assisting with antegrade therapies for benign biliary disease, the HG tract can also be used to aid in the diagnosis of suspected malignant biliary strictures. First, brushings can be performed with tissue sent for cytology and FISH. As reportedly previously, antegrade cholangioscopy can be also performed through the HG tract [3,13-15]. This enables direct visualization of strictures with acquisition of intraductal forceps biopsies. Cholangioscopy with intraductal biopsies has been shown to have a sensitivity of 60.1%, specificity of 98.0%, and diagnostic accuracy of 77% in the diagnosis of malignant biliary strictures [17,18].

While EUS-HG is now more widely used for biliary decompression in malignant obstruction, little is known about its potential impacts on surgery. A case series looking at EUS-CD for decompression as a bridge to surgery reported five cases of successful pylorus sparing Whipple procedure after EUS-CD [19]. However, there is only one case report that describes preoperative EUS-HG in a patient with Roux-en-Y gastric bypass anatomy and obstructive jaundice from a pancreatic head mass. EUS-HG was performed with successful biliary decompression prior to Whipple procedure [4]. 

## Conclusions

Our case series highlights the role of EUS-HG in suspected malignant biliary obstruction after unsuccessful ERCP. In each patient, successful biliary decompression was accomplished without adverse events. In two cases, cholangioscopy with biopsies and brushings for cytology and FISH helped in establishing a diagnosis of malignancy. In the third case, cold forceps biopsies demonstrated an ampullary adenoma with intraductal extension and hence diagnostic evaluation was not pursued. Lastly, EUS-HG did not impact surgical candidacy in any of the cases.

EUS-HG is a safe and effective alternative to PTBD for biliary decompression when ERCP is not feasible. In cases of suspected malignant biliary obstruction, it also enables further diagnostic evaluation to be performed and does not preclude future surgical options.
